# Latinx and White Adolescents’ Preferences for Latinx-Targeted Celebrity and Noncelebrity Food Advertisements: Experimental Survey Study

**DOI:** 10.2196/53188

**Published:** 2025-01-31

**Authors:** Marie A Bragg, Samina Lutfeali, Daniela Godoy Gabler, Diego A Quintana Licona, Jennifer L Harris

**Affiliations:** 1 Department of Population Health NYU Grossman School of Medicine New York, NY United States; 2 Food Environment and Policy Research Coalition NYU Grossman School of Medicine New York, NY United States; 3 Marketing Department Stanford Graduate School of Business Stanford University Stanford, CA United States; 4 Mailman School of Public Health Columbia University New York United States; 5 Rudd Center for Food Policy & Health University of Connecticut Hartford, CT United States

**Keywords:** Latinx, Hispanic, adolescents, marketing, celebrities, Spanish, advertisements, products, brands, food, unhealthy, beverages, diet, nutrition, consumers, intention, purchasing, attitudes, perceptions, preferences, youth

## Abstract

**Background:**

Exposure to food advertisements is a major driver of childhood obesity, and food companies disproportionately target Latinx youth with their least healthy products. This study assessed the effects of food and beverage advertisements featuring Latinx celebrities versus Latinx noncelebrities on Latinx and White adolescents.

**Objective:**

This web-based within-subjects study aims to assess the effects of food and beverage advertisements featuring Latinx celebrities versus Latinx noncelebrities on Latinx and White adolescents’ preferences for the advertisements and featured products.

**Methods:**

Participants (N=903) were selected from a volunteer sample of adolescents, aged 13-17 years, who self-identified as Latinx or White, had daily internet access, and could read and write in English. They participated in a web-based Qualtrics study where each participant viewed 8 advertisements for novel foods and beverages, including 4 advertisements that featured Latinx celebrities and the same 4 advertisements that featured Latinx noncelebrities (matched on all other attributes), in addition to 2 neutral advertisements (featuring bland, nontargeted products and did not feature people). Primary outcomes were participants’ ratings of 4 advertisements for food and beverage brands featuring a Latinx celebrity and the same 4 advertisements featuring a Latinx noncelebrity. Multilevel linear regression models compared the effects of celebrities and differences between Latinx and White participants on attitudes (advertisement likeability; positive affect; and brand perceptions) and behavioral intentions (consumption; social media engagement—“liking;” following; commenting; tagging a friend).

**Results:**

Latinx (n=436; 48.3%) and White (n=467; 51.7%) participants rated advertisements featuring Latinx celebrities more positively than advertisements featuring noncelebrities on attitude measures except negative affect (*Ps*≤.002), whereas only negative affect differed between Latinx and White participants. Two of the 5 behavioral intention measures differed by celebrity advertisement status (*P=*.02*; P*<.001). Additionally, the interaction between celebrity and participant ethnicity was significant for 4 behavioral intentions; Latinx, but not White, participants reported higher willingness to consume the product (*P*<.001), follow brands (*P*<.001), and tag friends (*P*<.001). While White and Latinx adolescents both reported higher likelihoods of “liking” advertisements on social media endorsed by Latinx celebrities versus noncelebrities, the effect was significantly larger among Latinx adolescents (*P*<.01).

**Conclusions:**

This study demonstrates the power of Latinx celebrities in appealing to both Latinx and White adolescents but may be particularly persuasive in shaping behavioral intentions among Latinx adolescents. These findings suggest an urgent need to reduce celebrity endorsements in ethnically targeted advertisements that promote unhealthy food products to communities disproportionately affected by obesity and diabetes. The food industry limits food advertising to children ages 12 years and younger, but industry self-regulatory efforts and policies should expand to include adolescents and address disproportionate marketing of unhealthy food to Latinx youth and celebrity endorsements of unhealthy products.

## Introduction

Developing obesity during adolescence increases future risk of diabetes, cardiovascular disease, and diet-related cancers during adulthood [1-7]. Youth of color experience higher rates of obesity relative to White youth [8-10]. More than 37% of Latinx adolescents are overweight or obese relative to 26.5% of White adolescents [1,11], indicating an urgent need to reduce rates of obesity among Latinx youth.

Food marketing is a major driver of adolescent and childhood obesity [12-16]. Food companies spend more than US $1.8 billion on youth-directed marketing [17,18], which primarily promotes products that are energy-dense and nutrient-poor [19-22]. Such exposure is concerning given the large body of evidence showing that viewing food advertising increases children’s preferences for promoted foods and brands, caloric intake, purchases, and requests for advertised products [2,16,23-26].

Adolescents and youth of color represent key consumer groups to food marketers not only because of their spending power [27] and trendsetting abilities [28,29] but also because of their unique responsiveness to advertising [19]. Compared to children and adults, adolescents are more impulsive and have lower inhibitory control [30]. They use brands to help elevate their feelings of self-worth [30], fit in with their desired peer group [31], and distinguish themselves from their caregivers [32]. Food and beverage brands aim to appeal to Latinx youth through targeted or multiethnic marketing, which refers to identifying a community that shares some common needs or characteristics that an organization—in this case, marketers—uses to appeal to that group [33]. While multiethnic advertising aims to reach consumers of diverse ethnicities, monoethnic advertising reaches members of a single ethnic group [33]. A study by Shao et al [34] found that socially distinctive consumers (eg, Latinx minorities) are more likely to prefer same-race monoethnic advertisements because this exposure promotes a sense of shared identity and belonging [34]. Janssen et al [35] explored advertising on Instagram and found that celebrity influencers with a high number of followers have more “likes” and more followers with positive attitudes toward the products they endorse; this results in greater purchase intention [35]. While targeted marketing is not inherently problematic, monoethnic advertising coupled with celebrity or influencer sponsorships can potentially target Latinx adolescents and Generation Z teens with health-harming products that may exacerbate health disparities.

Adolescents are exposed to food marketing on social media [36-38], but few studies have examined how adolescents respond to such marketing. One cross-sectional study showed that among a nationally representative sample of adolescents, more than 70% of them self-reported that they “liked,” shared, or followed food and beverage brand accounts [39]. The same study also found that Black and less acculturated Latinx adolescents were more likely to self-report that they engaged with brand accounts than White adolescents [39]. Recent experimental studies have revealed that adolescents rate social media advertisements highly when those advertisements have high numbers of “likes” [36]. One randomized trial examining the impact of influencer marketing—the promotion of products by popular web-based celebrities—showed that influencer marketing of unhealthy foods on social media significantly increased children’s consumption of unhealthy snacks [40,41].

Exposure to unhealthy food advertisements that feature celebrity influencers is concerning as it can capture adolescents’ attention through its appealing features. Viewing these advertisements can increase positive affect and feelings of connection to the celebrity or influencer because they are often seen as trustworthy and are already well-liked by the adolescent [42-44]. These positive feelings toward the celebrity or influencer then elicit positive feelings (greater positive affect, liking, and desire for the product) toward the unhealthy food and beverage they are promoting [45]. Previous studies have assessed participants’ responses to advertisements using 3 attitudinal ratings, including how much they like the advertisement, brand, and product, which allows researchers to compare such ratings across experimental conditions [46]. Such attitudinal ratings are measured through self-report questions that ask how much the participant likes the advertisement, how much they like the brand, and how much they like the product featured in the advertisement. Consequently, the likelihood of the adolescent engaging with the advertisement posts (eg, through “likes,” comments, and shares) increases, along with the likelihood that they will subsequently purchase or consume unhealthy food [42,47-51]. Purchase intentions are measured via a self-report question and defined as a participant’s willingness to purchase the product in the near future. Previous studies have used purchase intention questions as an outcome when objective measurements of purchasing behavior are not possible [52-57]. Previous research has measured and experimentally compared this engagement by participants with brands and advertisements on social media. Further, Social Norms Theory proposes that adolescents in particular, may be especially vulnerable to many of the racially targeted marketing practices that companies use on social media [58,59] because adolescents are highly sensitive to their peers [60,61], popularity cues, and conformity pressures [62,63].

Research has shown that these feelings of connection may be even stronger when the race of the person featured in the advertisement matches the race of the adolescent [42,64]. A number of theoretical frameworks in social psychology may inform an understanding of this effect. According to the social identity theory, individuals derive a significant portion of their self-concept from their group memberships, leading to the formation of social identities [65]. Emphasizing the importance of social categorization, social comparison, and group identification in shaping attitudes and behaviors, social identity theory suggests that Latinx adolescents’ ethnic identity may play a crucial role in shaping their self-concept. Racially targeted marketing that features Latinx celebrities can tap into this social identity, thereby fostering a sense of belonging and positively influencing attitudes toward the advertised product. Relatedly, Distinctiveness Theory may help explain the heightened appeal and influence of food advertisements featuring Latinx celebrities. Distinctiveness Theory explains that individuals define themselves based on rare traits in their environment [42,66]. Ethnicity in particular is an attribute related to distinctiveness and has been shown to hold greater significance in self-definition than other social categories [66]. Because Latinx adolescents are part of a distinctive minority group, distinctiveness theory suggests that they may be acutely attuned to and influenced by ethnically targeted marketing.

Despite the pervasiveness of targeted marketing, no experimental studies have examined the effects of Latinx-targeted food marketing on Latinx and White adolescents. This study assessed the effects of food and beverage advertisements featuring a Latinx celebrity on the same advertisements featuring a Latinx noncelebrity (ie, an individual who is not famous), including differential effects on Latinx and White adolescents. Examining the influence of celebrity-endorsed food advertisements on the preferences of Latinx and White youth will enhance the field’s theoretical understanding of how ethnically targeted marketing uniquely affects Latinx youth, which can help inform practical implications on how public policies can address unhealthy food marketing that targets youth of color. By harnessing a rigorous 2×2 mixed factorial design, this study is the first to objectively compare celebrities’ and noncelebrities’ food endorsements on Latinx and White youth. Our research questions, therefore, are as follows:

- To what extent does exposure to Latinx celebrities’ food advertisements increase adolescents’ preferences for the advertisement and the featured product, compared to Latinx-noncelebrities’ food advertisements?
- To what extent does exposure to Latinx people in food advertisements increase preferences for the advertisements among Latinx adolescents compared to White adolescents?

We predicted that (1) both Latinx and White adolescents would report higher preferences, including more positive attitudes (advertisement likeability; positive affect; and brand perceptions) and behavioral intentions (product consumption and social media engagement) when advertisements featured Latinx celebrities versus Latinx noncelebrities; (2) Latinx adolescents would report higher preferences for all advertisements compared to White adolescents; and (3) Latinx adolescents would show a stronger positive response to Latinx celebrity advertisements than White adolescents.

## Methods

### Study Design

This web-based study used a rigorous 2×2 mixed-factorial experimental design with 2 independent variables: the ethnicity of the participant (Latinx or White) and whether the person in the advertisement was a celebrity or noncelebrity. Latinx and White adolescent participants (aged 13-17 years) each viewed 8 advertisements for novel foods and beverages, including 4 advertisements that featured Latinx celebrities and the same 4 advertisements that featured Latinx noncelebrities (matched on all other attributes), in addition to 2 neutral advertisements. Novel food advertisements feature brands that participants are likely to be unfamiliar with, either created by researchers or brands from other countries. Using novel brands minimizes the likelihood that participants’ ratings will be driven by previous brand preferences and experiences. For example, using a well-known brand such as Coca Cola would drown out our ability to examine the effects of Latinx celebrities’ endorsements due to preexisting attitudes and preferences by consumers. After viewing each advertisement, participants provided attitude measures (for someone like me, advertisement likeability, positive and negative affect, and brand perceptions) and behavioral intentions (consumption, “liking,” following, commenting, and tagging a friend), before viewing the next advertisement. These outcome measures have been used in other food marketing research studies as primary and secondary outcomes [39,41,52].

### Participant Recruitment

In January 2019, we recruited participants through Dynata, a survey firm that maintains panels of thousands of adolescents and adults in the United States who are interested in completing surveys in exchange for incentives such as cash, prize drawings, or donations to charity. Dynata’s recruitment involves a rigorous process that uses multiple steps to recruit and screen potential participants. First, randomly selected participants from members of Dynata’s panels are combined with a pool of potential participants who are joining Dynata for the first time after responding to web-based recruitment materials. Those recruitment materials include banner advertisements on social media platforms and other commonly visited websites where participants might notice and respond to the flyers. This combined group of potential participants then receives an invitation to “take a survey,” but are not provided with additional details about surveys that might introduce selection bias (eg, “take a survey about food” might attract people with an interest in food). Second, to be eligible for a study, potential participants complete quality control questions and are randomly matched with surveys for which they likely qualify. Finally, among the randomly selected participants who expressed an interest in and have been randomly matched with a survey, a subset is randomized to complete a specific survey and then receives a link to the survey.

Pretest (N*=*50) and study participants (N*=*903) were adolescents, aged 13-17 years, who self-identified as Latinx, or White, had daily internet access, and could read and write in English. Of the 1509 eligible participants who responded to the survey, 992 participants (65.7%) completed all questions. An additional 89 participants did not answer 8 of 10 attention-check questions correctly and were excluded from the analyses. These attention-check questions asked, “What does this advertisement say?” with 3 multiple-choice answers. The final sample included 903 adolescents (436 (48.3%) Latinx adolescents and 467 (51.7%) White adolescents).

### Ethical Considerations

The Institutional Review Board for the New York University School of Medicine approved the study (protocol #115-0087), in accordance with all applicable regulations. Parents voluntarily provided consent and adolescent participants provided assent after reading general information about the study, explaining the nature and possible consequences of the study, and the length of the survey. Study data were deidentified to protect participants’ privacy and confidentiality. Participants were compensated by our recruitment firm, Dynata, with incentives such as cash, prize drawings, or donations to charity.

### Materials

To develop the 8 food and beverage advertisements, we first created pairs of advertisements for 8 novel foods and beverages using Photoshop (Adobe). Each pair included 1 advertisement that featured a Latinx celebrity and a matched advertisement with a Latinx noncelebrity. Because brand familiarity for well-known products (eg, Coca Cola) heavily influences advertisement ratings and the difficulty of changing preexisting attitudes about known brands [67], we selected brands that were only available in countries outside the United States. To increase the likelihood that the advertisements were perceived as Latinx-targeted and that participants would recognize the celebrities, the advertisements included a slogan with at least 1 Spanish word or the name of the celebrity in the slogan or as a signature.

The 16 advertisements (8 pairs) for pretesting included 4 male and 4 female celebrity or noncelebrity pairs and 4 foods and 4 beverages. We pretested these advertisements among 20 Latinx and White adolescents to match the advertisement pairs on the attractiveness of the person in the advertisement, perceived target audience, and celebrity recognition.

Pretest participants first rated the attractiveness of the person in the advertisement, indicated the perceived race or ethnicity of the person, and identified whether the person in the advertisement was a celebrity. They also indicated how much they thought the advertisement was intended for people like them and for people in their age group.

**Figure 1:**
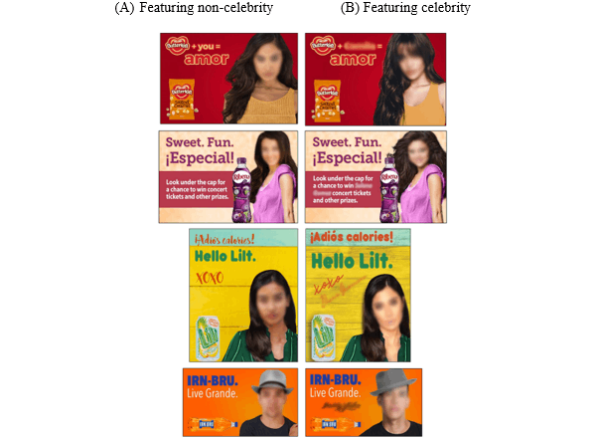
Final set of 4 pairs of advertising stimuli embedded into web-based survey. Each pair included (A) an advertisement featuring a Latinx noncelebrity and (B) an advertisement featuring a Latinx celebrity. Faces and names of the celebrities are blurred in the figure, but participants saw unblurred images.

We excluded 1 pair of advertisements because only 10% of participants (n=2) perceived the celebrity to be Latinx and another pair because the majority did not recognize the celebrity. We excluded 2 additional pairs because they scored the lowest on both celebrity recognition and attractiveness measures. The final set of 4 advertisement pairs (Figure 1) included 3 advertisement pairs that featured Latina women alongside a sugar-sweetened beverage (n=2 pairs) or unhealthy food product (n=1 pair) and one that featured a Latinx male alongside a sugar-sweetened beverage. Similar to previous research, we blurred the faces and names of the celebrities in the advertisements in Figure 1. The participants saw unblurred images [35,68].

**Figure 2:**
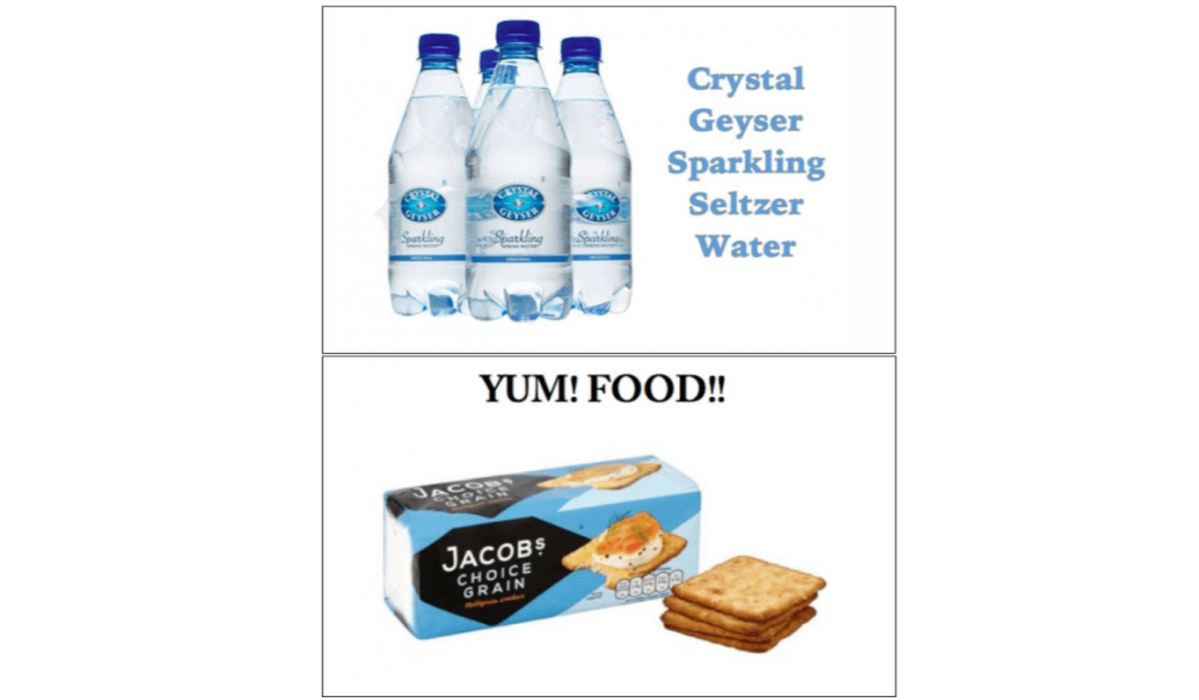
Final two 2 neutral, nontargeted advertising stimuli (control) embedded into web-based survey.

We also used Photoshop to create 2 additional advertisements to serve as neutral nontargeted stimuli. These 2 advertisements featured bland, unsavory products (ie, water and saltine crackers) and did not show people (Figure 2).

They were used to compare advertisement ratings by Latinx and White adolescents to enable us to control for overall differences in advertisement ratings in our final models.

### Survey Procedures

The web-based survey was designed using Silver Lake and CPP Investment’s Qualtrics software, and the functionality of the survey was tested by the investigators before launching the study. Participants were required to complete the survey on a computer (instead of a mobile device) to ensure the visibility of advertisements. Participating on a mobile device might have made it more difficult to view the features of the advertisements in the survey due to the smaller screen size compared to desktops. Median survey completion time was 23 minutes. The study setting was anywhere a participant could access a computer to complete the experimental survey.

After providing assent and answering eligibility questions, participants were told the following cover story to conceal the true purpose of the study: “We are working with MTV to select commercials that their viewers might like and are interested in your attitudes and opinions on products featured in these advertisements. Participation in this study will involve viewing several different food and beverage advertisements and answering questions about them.” All participants viewed and rated each of the 10 advertisements in a specific order as follows: they first viewed the neutral advertisement for water; then 2 celebrity advertisements and 2 noncelebrity advertisements for different brands presented in random order; then viewed the neutral advertisement for crackers; and finally, the remaining celebrity and noncelebrity advertisements presented in random order. This order ensured that participants did not see advertisements for 2 brands in the same group of 4 advertisements.

### Primary Outcome Measures

The primary outcome measures and dependent variables are listed in Table 1.

**Table 1:** Primary outcome measures for cognitive responses to advertisements, advertisement efficacy, and behavioral intentions after viewing advertisements.

Outcomes			Survey questions
**Advertisement rating questions: Cognitive responses to advertisements and advertisement efficacy**			
	Likeability^a^	“How much did you like this ad?” (0=“strongly disliked it” to 100=“liked it a lot”) [16,36]	
	Positive affect	“Did the ad for [Coke] make you feel: cheerful; good; pleased; stimulated; soothed?” (0=“not at all” to 100=“very much so”. Scores were averaged into a composite positive affect score [69].	
	Negative affect	“Did the ad for [Coke] make you feel: insulted; irritated; repulsed?” (0=“not at all” to 100=“very much so”. Scores were averaged into a composite negative affect score [69].	
	Taste preference	“How much do you think you would like the taste of this product” (0=“Not at all” to 100=“A lot”) [53].	
	Advertisement efficacy^b^	“I like the brand in this ad,” “I react favorably to the brand in this ad,” “The brand in this ad is appealing,” “The brand in this ad is good,” and “The brand in this ad is pleasant,” (0=“disagree” to 100=“agree”) [70]. Brand liking statements were averaged for a combined brand perception rating.	
**Behavioral intention questions**			
	Likelihood to consume the advertised brand	“How likely would you be to consume this product if it was offered to you?” (0=“very unlikely” to 100=“very likely”) [71]	
	Likelihood of following or liking the brand on social media^a^	“What is the likelihood you would Follow or Like this brand on Facebook, Instagram, Twitter, Snapchat, or Tumblr?” (0=“very unlikely” to 100=“very likely”) [16,36]	
	Willingness to engage with the brand or advertisement on social media^a^	How likely would you be to engage with this brand on social media?: “I would comment on the brand’s post” (0=“very unlikely” to 100=“very likely”) ; “I would ”Like“ this brand’s post” (0=“very unlikely” to 100=“very likely”) ; “I would tag a friend” (0=“very unlikely” to 100=“very likely”) [16,36]	
	Likelihood they were the intended audience for the post	“I feel the advertisement was intended for people like me” (0=“disagree” to 100=“agree”); “I do not believe the company was targeting consumers like me” (0=“disagree” to 100=“agree”); “The advertiser made this advertisement for people like me.” (0=“disagree” to 100=“agree”). The inverse of question 2 was calculated and the scores of the 3 questions were averaged into a composite score [72].	

^a^This item has not been validated but has been used before in previous research [16,36].

^b^This item is based on a published commonly used marketing scale study but was adapted [70].

Participants provided their attitudes about advertisements and brands immediately after viewing each advertisement, before viewing the next advertisement, using a sliding scale (0-100) for all questions (Table 1). We used 0-100 response scales to minimize bias and lack of reliability versus Likert scales [73], and, among some participant populations (eg, under-resourced communities), Likert scales contradict other response formats [74]. Other studies have shown that 0-100 scales offer stronger psychometric properties than Likert scales [75].

The question, “How much did you like this ad?” (0=“strongly disliked it” to 100=“liked it a lot”) assessed advertisement likeability. A commonly used and validated marketing survey was used to evaluate affective advertisement responses [69]. Participants responded to the following 8 prompts: “Did the ad for [Coke] make you feel…cheerful; good; pleased; stimulated (positive affect); soothed; insulted; irritated; repulsed (negative affect)?” Affective advertisement responses included positive (cheerful, good, pleased, stimulated, and soothed) and negative (insulted, irritated, and repulsed) attributes, rated as 0=“not at all” to 100=“very much so.” Positive and negative attributes were averaged into separate composite positive and negative affect scores. Taste preference was measured with 1 question: ”How much do you think you would like the taste of this product” (0=“Not at all” to 100=“A lot”) [69]. Advertisement efficacy was measured with an adapted version of a commonly used marketing scale that asked participants the following 5 questions: “I like the brand in this ad,” “I react favorably to the brand in this ad,” “The brand in this ad is appealing,” “The brand in this ad is good,” and “The brand in this ad is pleasant,” and rated from 0=“disagree” to 100=“agree” [71]. Brand liking statements were averaged for a combined brand perception rating [70].

**Figure 3:**
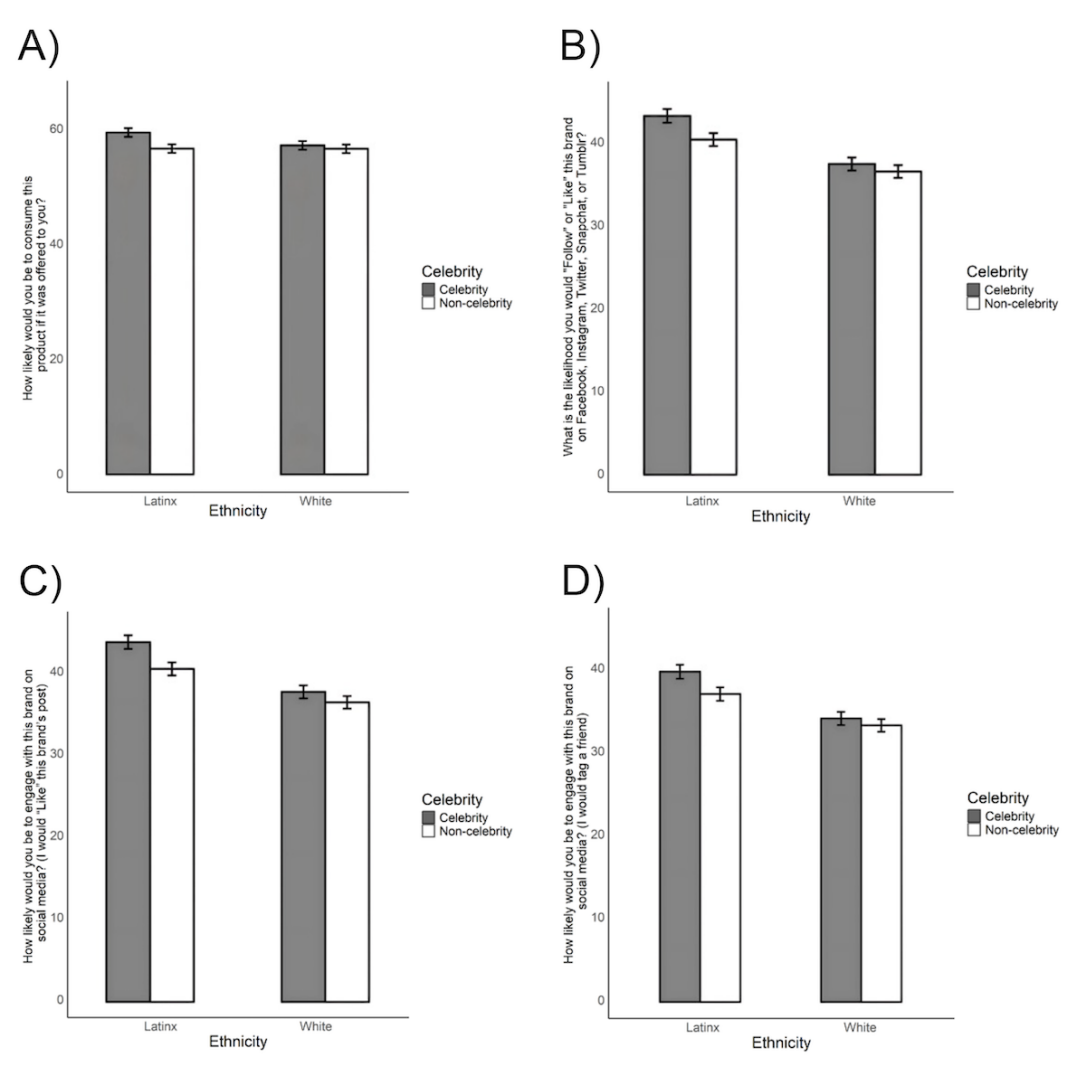
Latinx (n=436) and White (n=467) adolescents’ ratings of likelihood of (A) consuming the product if it was offered to them; (B) following or liking the brand on Facebook, Instagram, Twitter, Snapchat, or Tumblr; (C) “Liking” the post on social media; and (D) tagging a friend on social media. Error bars indicate SDs.

Behavioral intention measures included the likelihood to consume the advertised brand [71] and willingness to engage with the brand or advertisement on social media, including the following questions: “What is the likelihood you would Follow or Like this brand on Facebook, Instagram, Twitter, Snapchat, or Tumblr?”; How likely would you be to engage with this brand on social media? (“I would comment on the brand’s post” [0=“very unlikely” to 100=“very likely”]; “I would “Like” this brand’s post” [0=“very unlikely” to 100=“very likely”]; “I would tag a friend” [0=“very unlikely” to 100=“very likely”]). The willingness to engage measures have not been validated but have been used before in previous research [16,36]. All responses were assessed on a sliding scale from 0=“very unlikely” to 100=“very likely” (Figure 3).

Participants rated the likelihood they were the intended audience for the post across all 8 advertisements that featured people, using a scale developed by Aaker et al [72]. The scale asked three questions: (1) “I feel the advertisement was intended for people like me,” (2) “I do not believe the company was targeting consumers like me,” and 3) “The advertiser made this advertisement for people like me.” We assessed these responses using a sliding scale from 0=“disagree” to 100=“agree.” We reverse-coded question 2 and then averaged the scores.

### Other Measures

Participants provided demographic characteristics, including age, race, ethnicity, grade level, parental education, and height and weight. They then indicated how often they checked social media if they had access to a smartphone, and which social media platforms they used. Participants who identified as Latinx also completed the Multigroup Ethnic Identity Measure-Revised, a 6-item measure that assesses exploration of and commitment to ethnic identity on a 5-point Likert scale: 1=strongly disagree to 5=strongly agree. The overall composite Multigroup Ethnic Identity Measure-Revised score is calculated by averaging the scores for each of the 6 items. The scale has been validated among adolescents to measure ethnic identity (among Latinx adolescents, α=.930) [76-78].

### Statistical Analysis

We used 2-tailed *t* tests (continuous variables) and chi-square tests (categorical variables) to compare the demographic characteristics of Latinx and White participants. We also compared Latinx and White participants’ ratings of the 2 neutral advertisements on all outcomes to determine whether the 2 groups systematically rated advertisements differently on any of the measures.

We used multilevel linear regression to account for multiple ratings by each participant. Dependent variables included all attitude and behavioral measures. Independent variables included binary terms for participant ethnicity and presence of a celebrity in the advertisement, an interaction term between participant ethnicity and celebrity, random effect of participant, and categorical brand indicator. There were no differences in Latinx and White adolescents’ ratings of the neutral advertisements that did not feature people, so we did not control for that variable in the model. Across all the outcomes except targetedness, Latinx participants did not rate the neutral advertisements any differently than the White participants. They differed on targetedness—Latinx participants said they thought the neutral advertisements were more targeted to them. Due to differences in age by participant ethnicity (*P=.*04), we included a categorical indicator of age as a covariate in all models. All analyses were conducted in R (R Foundation for Statistical Computing) in February 2020.

## Results

### Overview

More than half of participants (n=540, 59.8%) were aged 13-15 years and 40.2% (n=363) were aged 16-17 years (Table 2). Approximately one-half identified as Latinx (n=436, 48.3%). There were no significant differences in parental education (*P*=.21) or participant sex (*P*=.13) between Latinx and White participants. The majority of participants (n=870, 96.3%) reported having one or more social media accounts, and 41.1% reported using a smartphone “almost constantly” to access social media.

**Table 2:** Sociodemographic and behavioral characteristics of the study sample (N=903) and self-reported social media use survey responses.

				Total sample (N=903)		Latinx adolescents (n=436)		White adolescents (n=467)		*P* values for chi-squared tests^a^	
**Sociodemographic characteristics**											
	**Age^b^ (years), n (%)**										.04
		13-15	540 (59.8)		276 (63.3)		264 (56.5)				
		16-17	363 (40.2)		160 (36.7)		203 (43.5)				
	**Highest parent educational level**^c^, **n (%)**										.21
		High school, GED^d^, or lower	191 (21.2)		81 (18.6)		110 (23.6)				
		Vocational school or some college	327 (36.2)		167 (38.3)		160 (34.3)				
		Bachelor degree	239 (26.5)		111 (25.5)		128 (27.4)				
		Master degree or advanced graduate work	138 (15.3)		71 (16.3)		67 (14.4)				
		Not sure or unknown	8 (0.9)		6(1.4)		2 (0.4)				
	**Sex, n (%)**										.13
		Male	148 (16.4)		79 (18.1)		69 (14.8)				
		Female	753 (83.4)		355 (81.4)		398 (85.2)				
		Nonbinary or other	2 (0.2)		2 (0.5)		0 (0.0)				
	MEIM-R^e^ (α=.930), n (%)		N/A^f^		60.23 (24.3)		N/A				
	Less than or equal to median (60.8), n (%)		N/A		219 (50.2)		N/A				
	Greater than median (60.8)), n (%)		N/A		217 (49.8)		N/A				
**Behavioral characteristics**											
	**How often do you use your smartphone to access social media accounts, such as Facebook, Instagram, and Snapchat, each day?^c^, n (%)**										.07
		Almost constantly	371 (41.1)		193 (44.3)		178 (38.1)				
		Several times a day	354 (39.2)		169 (38.8)		185 (39.6)				
		Once a day or less frequently	178 (19.7)		74 (16.97)		104 (22.3)				
	**Social media access on smartphones^c^, n (%)**										.12
		I have my own smartphone	833 (92.3)		409 (93.8)		424 (90.8)				
		I do not have my own smartphone	70 (7.8)		27 (6.2)		43 (9.2)				
	**Which of the following social media accounts do you have?, n (%)**										
		Facebook^b^	644 (71.3)		296 (67.9)		348 (74.5)				
		Instagram^b^	650 (71.98)		334 (76.6)		316 (67.7)				
		Snapchat^b^	601 (66.6)		325 (74.5)		276 (59.1)				
		Twitter	308 (34.1)		147 (33.7)		161 (34.5)				
		Tumblr	66 (7.3)		37 (8.5)		29 (6.2)				
		Other	14 (1.6)		6 (1.4)		8 (1.7)				
	**How many active social media accounts do you have?^b^, n (%)**										.10
		0	33 (3.1)		11 (2.5)		22 (4.7)				
		1	177 (19.8)		75 (17.2)		102 (21.8)				
		2	228 (24.8)		110 (25.2)		118 (25.3)				
		3	254 (28.0)		136 (31.2)		118 (25.3)				
		4	171 (18.9)		80 (18.4)		91 (19.5)				
		5	36 (3.99)		22 (5.1)		14 (3)				
		6	4 (0.4)		2 (0.4)		2 (0.4)				
	Average number of social media accounts, mean (SD)^b^		2.53 (1.2)		2.63 (1.2)		2.44 (1.3)		.02		
	Correctly identified celebrity status, (N=7224, 8 advertisements per person)^b^, n (%)		5006 (69.3)		2572 (73.4)		2434 (65.2)		<.001		

^a^*P* values for chi-square tests comparing the demographic data between Latinx and White adolescents.

^b^*P*<.05 comparing distributions between Latinx and White adolescents.

^c^.05<*P*<.25 comparing distributions between Latinx and White adolescents.

^d^GED: General Educational Development.

^e^MEIM-R: Multigroup Ethnic Identity Measure-Revised.

^f^N/A: not applicable.

### Attitudes About the Advertisement and the Brand

On average, participants reported liking the 8 advertisements that featured people (mean 53.58, SD 28.70; Table 3).

Positive affective responses were rated neutrally (mean 49.44, SD 28.85), while participants reported overall low negative affective responses to advertisements (mean 18.77, SD 25.79). Participants rated their perception of the brand on average as 51.63 (SD 29.25) across all 8 advertisements that featured people.

For all attitude measures except negative affect, all participants rated advertisements significantly more positively for advertisements with celebrities than with noncelebrities; there were no main effects of participant ethnicity nor interactions between celebrity and participant ethnicity. However, Latinx participants reported significantly more negative affect toward all advertisements (*P*=.01).

For brand perception, we found a main effect of celebrity but did not find an interaction, indicating that Latinx and White adolescents rated the brands with celebrities significantly higher than the brands without celebrities.

**Table 3:** Latinx (n=436) and White (n=467) adolescents’ ratings of attitudes toward food advertisements featuring Latinx celebrities and Latinx noncelebrities, and behavioral intentions postadvertisement exposure^a,b^.

Outcome			Latinx participants (n=436)				White participants (n=467)				Main effect of celebrity in the advertisement		Main effect of participant ethnicity		Interaction (participant ethnicity and celebrity in advertisement)
			Celebrity, mean (SD)		Noncelebrity, mean (SD)		Celebrity, mean (SD)		Noncelebrity, mean (SD)		β (*P* value)		β (*P* value)		β (*P* value)
Intended audience (targeting)			58.71 (0.49)		56.51 (0.47)		55.51 (0.51)		55.48 (0.50)		.03 (.95)		1.12 (.33)		2.17 (<.001)
**Attitudes**															
	How much did you like or dislike the ad?	55.53 (0.69)		52.72 (0.67)		53.85 (0.68)		52.30 (0.66)		2.15 (<.001)		1.22 (.44)		—^c^	
	Positive affective response	51.52 (0.68)		48.93 (0.67)		49.45 (0.69)		47.96 (0.67)		2.02 (<.001)		1.66 (.32)		—	
	Negative affective response	20.69 (0.66)		20.69 (0.64)		17.05 (0.57)		16.58 (0.55)		0.24 (.46)		3.77 (.01)		—	
	How much do you think you would like the taste of this product?	57.15 (0.71)		55.65 (0.71)		55.49 (0.72)		54.73 (0.71)		1.12 (.01)		1.42 (.38)		—	
	Brand perception	53.55 (0.69)		50.94 (0.68)		51.54 (0.70)		50.59 (0.69)		0.96 (.07)		0.52 (.76)		1.66 (.03)	
**Behavioral intentions**															
	How likely would you be to consume this product if it was offered to you?	58.88 (0.73)		56.12 (0.73)		56.67 (0.75)		56.09 (0.73)		0.59 (.36)		0.13 (.94)		2.17 (.02)	
	What is the likelihood you would “Follow” or “Like” this brand on Facebook, Instagram, Twitter, Snapchat, or Tumblr?	43.23 (0.82)		40.38 (0.78)		37.44 (0.78)		36.53 (0.76)		0.92 (.08)		4.01 (.049)		1.94 (.01)	
**How likely would you be to engage with this brand on social media?**															
	I would comment on the brand’s post	40.13 (0.81)		37.85 (0.79)		35.74 (0.78)		34.88 (0.76)		1.54 (.01)		3.79 (.06)		—	
	I would “Like” this brand’s post	43.91 (0.82)		40.65 (0.79)		37.84 (0.80)		36.56 (0.77)		1.28 (.02)		4.27 (.04)		1.98 (.01)	
	I would tag a friend	39.28 (0.83)		36.63 (0.80)		33.71 (0.79)		32.90 (0.76)		0.81 (.12)		3.92 (.06)		1.85 (.01)	

^a^All models included age as a covariate.

^b^All response items were on a scale ranging from 0 to 100.

^c^Not available.

### Behavioral Intentions

Participants reported that they would likely consume the product (mean 56.92, SD 31.22), and there was no main effect of celebrity or participant ethnicity. However, we found a significant interaction between participant ethnicity and celebrity (Table 3). Only Latinx participants indicated significantly higher consumption intention when the advertisement featured a celebrity compared to advertisements featuring a noncelebrity (*P*<.001).

On measures of social media engagement, participants rated their likelihood of following the brand on average 39.31 (SD 33.51) across all 8 advertisements. They indicated a similar likelihood to comment on the social media post (mean 37.09; SD 33.34), “like” the post (mean 39.65, SD 33.81), and “tag” a friend in the post (mean 35.55, SD 33.88).

The main effect of celebrity was significant for two of the four social media engagement measures: (1) participants were more likely to comment on or “Like” posts that featured celebrities in their advertisements compared to posts that featured noncelebrities (Table 3); (2) Latinx participants were more likely to “Follow” or “Like” brands or posts than White participants. There was a main effect of celebrity on participant’s likelihood of commenting on the post such that participants reported a higher likelihood of commenting on posts that featured celebrities (mean 38.1, SE 1.04 compared to those without (mean 36.5, SE 1.04; β=1.54; t_6320_=4.25; *P*<.001). We also found the main effects of celebrity (β=1.28; t_6319_=2.38; *P*=.02) and participant ethnicity (β=4.27; t_968_=2.09; *P*=.04) for willingness to “like” the brand. The main effects were qualified by an interaction (β=1.98; t_6319_=2.57; *P*=.01). While White participants reported higher willingness to “like” advertisements with celebrities compared to noncelebrities (mean 36.7, SE 1.42 vs mean 38.0, SE 1.42; *z*=2.38; *P*=.02); the effect of celebrity was significantly greater among Latinx participants (mean 41.0, SE 1.49 vs mean 44.3, SE 1.49; *z*=5.87; *P*<.001).

For willingness to “tag” a brand, we found no main effects of celebrity (t_6319_=1.55; *P*=.12) or participant ethnicity (t_963_=1.91; *P*=.06). However, we did find a significant interaction (t_6319_=2.46; *P*=.01). While White participants were equally likely to “tag” an advertisement with a celebrity (mean 33.9, SE 1.43) compared with an advertisement without a celebrity (mean 33.1, SE 1.43; *z*=1.55; *P*=.12), Latinx participants were more likely to “tag” an advertisement with a celebrity (mean 39.7, SE 1.50) compared to an advertisement without a celebrity (mean 37.0, SE 1.50; *z*=4.91; *P*<.001).

Finally, for willingness to follow a brand on social media, we found no main effect of celebrity (t_6319_=1.76; *P*=.08), but did find evidence of a main effect of participant ethnicity (β=4.01; t_964_=1.98; *P*=.05), as well as a significant interaction (β=1.94; t_6319_=2.59; *P*=.01). White adolescents reported the same willingness to follow a brand on social media regardless of whether the advertisement featured a celebrity (mean 37.6, SE 1.42) or not (mean 36.7, SE 1.42; *P*=.08). However, Latinx participants reported higher likelihood of following the brand on social media when the advertisement featured a celebrity (mean 43.6, SE 1.48 vs mean 40.7, SE 1.48; *z*=5.30; *P*<.001).

## Discussion

### Principal Findings

Our hypothesis for research question 1, is that both Latinx and White adolescents would report higher preferences, including more positive attitudes (advertisement likeability; positive affect; and brand perceptions) and behavioral intentions (product consumption; social media engagement), when advertisements featured Latinx celebrities versus Latinx noncelebrities was mostly supported. Specifically, both Latinx and White participants in our sample reported significantly higher positive attitudes and brand perceptions for brands endorsed by Latinx celebrities compared to Latinx noncelebrities. However, we found significant main effects of celebrity advertisements for just 2 behavioral intentions: likelihood to comment and like brand posts. The findings that celebrities increased the appeal of the advertisements and products reinforces previous research that showed celebrity-endorsed products are more appealing to adolescents [41] and preadolescents [79] compared to products endorsed by noncelebrities.

Our hypothesis for research question 2, that Latinx participants would report more positive attitudes and behavioral intentions for both Latinx celebrity and Latinx noncelebrity advertisements compared to White participants was supported for just 2 outcomes: likelihood to follow brands on social media and like a brand post. We found no significant main effects of participant ethnicity on positive attitudes, brand perceptions, or intent to consume. However, we did find the hypothesized interaction between celebrity and participant ethnicity for most behavioral intentions. Relative to White adolescents, Latinx adolescents reported a higher willingness to “like” and follow brands that featured celebrities compared to brands that featured noncelebrities. In addition, Latinx adolescents reported higher consumption intentions and likelihood to tag a post when advertisements featured celebrities compared to noncelebrities.

These results reinforce other studies on the effects of celebrity endorsements and targeted food marketing. Our findings that both Latinx and White adolescents reported a higher likelihood to comment, “like,” or tag a friend in the post on social media when the advertisements featured celebrities versus noncelebrities is similar to previous research showing that celebrity endorsements increase willingness to engage with brands [80]. Like other studies demonstrating that celebrity endorsements made adolescents more likely to choose the endorsed product [79], we identified a main effect of celebrity on adolescents’ willingness to consume the product—but only for Latinx adolescents.

Latinx celebrities may disproportionately influence Latinx adolescents’ behavioral intentions, including intent to consume and follow brands on social media, compared to White adolescents and noncelebrity advertisements. One potential explanation for the disproportionate influence of Latinx celebrities on Latinx youth is that Latinx communities are underrepresented in popular media, and positive portrayals in the media may heighten Latinx adolescents’ responses to targeted advertisements. Future studies should examine the extent to which exposure to Latinx-targeted advertisements increases Latinx adolescents’ actual food purchases and caloric consumption.

This study makes several contributions to the literature on the effects of food advertising and exposure to racially targeted food advertising. First, this is the first experimental study to examine the intersection of food advertising and celebrity endorsements among Latinx youth. The results indicate a direct causal effect of celebrities on willingness to consume the product and follow or “like” the brand on social media for both Latinx and White youth, even though—after controlling for other possible explanations—Latinx youth were more likely to believe that the advertisements with Latinx celebrities targeted them. These findings demonstrate the power of celebrity endorsements on adolescents’ preferences. Second, this study demonstrates that the intersection between celebrity endorsements and Latinx ethnicity is complex and warrants further research to disentangle these effects. Celebrities influenced adolescents’ attitudes about the advertisements, but it appears that Latinx celebrities may have a disproportionate influence on Latinx adolescents’ behavioral intentions.

### Limitations

This study has several limitations. First, although we only included brands from outside the United States to avoid brand familiarity potentially influencing outcomes, our pretest did not assess brand familiarity among participants. Some adolescents may have been familiar with the international brands in our advertisements, thereby influencing their ratings. An additional limitation is that we only included Latinx celebrities and Latinx noncelebrities. The lack of White people in advertisements limits our ability to discern if our outcomes were shaped by ethnic congruity or some other factor. Another limitation is that in using Dynata for participant recruitment, our sample was not nationally representative. Participants in Dynata’s sample are more likely to use the internet than the general US population, but since our research studies social media, this sample may better reflect our population of interest. Another limitation of opt-in panels is the risk of response bias (eg, providing socially desirable answers). However, we believe this risk is greatly reduced because the survey responses are anonymous, and research involving Dynata shows that adolescents express a variety of preferences (ie, they do not give socially desirable answers) [52-57]. Another limitation is that within-subjects study designs introduce possible “order effects” bias. Because our participants saw each advertisement (eg, the popcorn advertisement featuring a celebrity) and later saw its counterpart (eg, popcorn advertisement featuring a noncelebrity), order effects bias would suggest that the ratings of the second advertisement in a given pair could be affected by their reaction to the first advertisement in that pair. We aimed to reduce such bias, however, by (1) separating the presentation order of advertisements so that participants never saw two similar advertisements in a row; and (2) randomizing the order (eg, for each advertisement pair, some participants saw the celebrity advertisement first, whereas others saw the noncelebrity advertisement first). Although we would have preferred to avoid any possibility of order effects by using randomization via a between-subjects design, within-subjects is valuable when large sample sizes are not feasible—when designing the study, we knew we would not be able to recruit a large enough sample of Latinx adolescents to warrant a between-subjects design with multiple study conditions. Within-subjects’ designs are commonly used in experimental research studies, including studies focused on food-related behaviors [81-83]. Finally, we did not assess participants’ familiarity with the people featured in the advertisements, nor did we ask how much they liked the person in the advertisement. Both of those ratings should have been controlled in our analyses because they could have affected participants’ ratings. Our study has major strengths, though, including the tightly controlled manipulation of images and the pretesting of those matched celebrities and noncelebrities on perceived attractiveness and target audience. Research on the effects of monoethnic and celebrity advertising effectiveness on the Generation Z cohort should also be explored.

### Conclusions

Our data advance the theoretical understanding of targeting marketing by showing that viewing ethnicity-congruent food advertisements is particularly powerful for Latinx youth, which is concerning given Latinx communities experience high rates of diet-related diseases. These findings can help support policies that address unhealthy food marketing that targets communities of color, similar to how tobacco research on targeted marketing helped shed light on tobacco companies’ targeted marketing practices [84-87]. Furthermore, in response to concerns about targeted marketing, Facebook now prohibits any advertisement from targeting users based on race [88]. New York State’s proposed Predatory Marketing Prevention Act would aim to prevent advertisements from misleading consumers. These findings can be communicated to policymakers to consider including racial/ethnic-targeted marketing in such bills [89]. These findings suggest an urgent need to reduce celebrity endorsements in ethnically targeted advertisements that promote unhealthy food products to communities disproportionately affected by obesity and diabetes. Celebrities should consider choosing endorsements that support the health of their young fans, and powerful celebrities may be able to encourage companies to allow them to endorse healthier products. The food industry limits food advertising to children aged 12 years and younger, but our findings indicate that industry self-regulatory efforts should expand to include adolescents and address disproportionate advertising of unhealthy food to Latinx youth.

## Data Availability

The data generated during and/or analyzed during this study are available from the corresponding author on reasonable request.
